# Acquisition of microbiota according to the type of birth: an integrative review

**DOI:** 10.1590/1518.8345.4466.3446

**Published:** 2021-07-19

**Authors:** Gabriela Diniz Pinto Coelho, Lilian Fernandes Arial Ayres, Daniela Sezilio Barreto, Bruno David Henriques, Mara Rúbia Maciel Cardoso Prado, Camila Mendes Dos Passos

**Affiliations:** 1 Universidade Federal de Viçosa, Departamento de Medicina e Enfermagem, Viçosa, MG, Brasil.

**Keywords:** Microbiota, Obstetric Delivery, Cesarean Section, Parturition, Immune System, Newborn Infant, Microbiota, Parto Obstétrico, Cesárea, Parto, Sistema Imunitário, Recém-Nascido, Microbiota, Parto Obstétrico, Cesárea, Parto, Sistema Inmunológico, Recién Nacido

## Abstract

**Objective:**

to analyze scientific evidence regarding the relationship between the type of birth and the microbiota acquired by newborns.

**Method:**

this integrative review addresses the role of the type of delivery on newborns’ microbial colonization. A search was conducted in the Medical Literature Analysis and Retrieval System Online/PubMed and Virtual Health Library databases using the descriptors provided by Medical Subject Headings (MeSH) and Health Science Descriptors (DeCS).

**Results:**

infants born vaginally presented a greater concentration of *Bacteroides*, *Bifidobacteria*, and *Lactobacillus* in the first days of life and more significant microbial variability in the following weeks. The microbiome of infants born via C-section is similar to the maternal skin and the hospital setting and less diverse, mainly composed of *Staphylococcus*, *Streptococcus*, and *Clostridium*.

**Conclusion:**

the maternal vaginal microbiota provides newborns with a greater variety of colonizing microorganisms responsible for boosting and preparing the immune system. Vaginal birth is the ideal birth route, and C-sections should only be performed when there are medical indications.

## Abstract (pt)

**Objective:**

analisar as evidências científicas existentes na literatura sobre a relação da via de nascimento com a microbiota adquirida pelo recém-nascido.

**Método:**

trata-se de uma revisão integrativa sobre a influência da via de nascimento na colonização microbiótica no recém-nascido. Foi realizada uma busca na literatura por meio das bases de dados *Medical Literature Analysis and Retrieval System Online*/ PubMed e Biblioteca Virtual em Saúde, tendo como estratégia de busca a seleção de artigos baseados nos descritores desenvolvidos com *Medical Subject Headings* (termos MeSH) ou Descritores em Ciência da Saúde (DeCS).

**Resultados:**

os recém-nascidos por via vaginal apresentam nos primeiros dias de vida maior concentração de *Bacteroides, Bifidobacterias e Lactobacillus* e, com o passar das semanas, mostram maior variabilidade microbiótica. Os recém-nascidos por cesárea apresentam microbioma semelhante ao da pele materna e do ambiente hospitalar e possuem menor diversidade, sendo, principalmente, constituído de *Staphylococcus, Streptococcus e Clostridium*.

**Conclusão:**

a microbiota vaginal materna dispõe de uma maior variedade de microrganismos colonizadores, os quais são responsáveis por auxiliar na capacitação e melhor adequação ao sistema imunológico do recém-nato. Evidencia-se que o parto vaginal é a via ideal, ou seja, a cesariana deve ser realizada apenas quando existem indicações reais.

## Abstract (es)

**Objetivo:**

analizar las evidencias científicas existentes en la literatura sobre la relación de la vía de nacimiento con la microbiota adquirida por el recién nacido.

**Método:**

se trata de una revisión integradora sobre la influencia de la vía de nacimiento en la colonización de la microbiota en el recién nacido. Fue realizada una búsqueda en la literatura en las bases de datos *Medical Literature Analysis and Retrieval System Online*/PubMed y Biblioteca Virtual en Salud, teniendo como estrategia de búsqueda la selección de artículos basados en los descriptores desarrollados en el *Medical Subject Headings* (términos MeSH) o Descriptores en Ciencia de la Salud (DeCS).

**Resultados:**

los recién nacidos por vía vaginal presentan, en los primeros días de vida, mayor concentración de *Bacteroides, Bifidobacterias y Lactobacillus*; y, con el pasar de las semanas muestran mayor variabilidad de la microbiota. Los recién nacidos por cesárea presentan microbioma semejante a la piel materna y al ambiente hospitalario, poseyendo menor diversidad y siendo principalmente constituida de *Staphylococcus, Streptococcus y Clostridium*.

**Conclusión:**

la microbiota vaginal materna proporciona al neonato una mayor variedad de microorganismos colonizadores que son responsables por auxiliar en la capacitación y mejor adecuación de su sistema inmunológico. Se evidencia que el parto vaginal es la vía ideal y que la cesárea debe ser realizada apenas cuando existen indicaciones reales.

## Introduction

The Brazilian obstetrical context is of concern and considered a public health problem due to various factors, including the C-section epidemic. According to the World Health Organization (WHO), the rate of C-sections should not exceed 15%^1^; however, approximately 90% of the deliveries in the supplementary health system are C-sections, while in the Unified Health System (SUS), this rate reaches 45%^2^.

There is a higher risk of bleeding in the intra- and post-operative of C-sections^3^^-^ 5, in addition to infections in the surgical site^6^^-^ 7, puerperal sepsis, deep venous thrombosis, endotoxin shock^8^, and lengthier hospitalizations due to slower recovery^6^.

The route of birth delivery may also influence a newborn’s health. Children born via C-section are at increased risk of developing asthma, systemic connective tissue disorders, juvenile arthritis, inflammatory bowel disease, immune deficiencies, and leukemia^9^. Part of these diseases is believed to be related to the maturation of the neonatal immune system^10^^-^ 11.

Studies suggest that the immune system of a newborn is widely stimulated when first exposed to microorganisms during neonatal life^11^, while the type of delivery shapes an infant’s microbial communities, which consequently play a role in his/her immune system maturation^12^.

Therefore, we propose that the route of birth delivery influences the colonization of microorganisms in newborns. Nonetheless, studies seldom address the mechanisms involved in this adaptation according to the type of birth. This study’s findings are expected to support the choice of the type of birth, decreasing unnecessary C-sections without a medical indication. Fetal morbidity and mortality caused by C-sections and inappropriate adaptation of the neonatal immunological system are also expected to decrease.

Therefore, this study’s objective was to analyze scientific evidence concerning the relationship between the route of birth delivery and the newborns’ acquisition of microbiota.

## Method

This is an integrative review, which can be defined as a method capable of synthesizing scientific knowledge regarding a given problem^13^. It also allows researchers to monitor a topic’s development over time and formulate new theories and generate knowledge^14^. It allows incorporating studies with the most diverse methodological designs, and for this reason, is considered a complex tool^14^.

This study strictly followed the proposed stages^15^, namely: 1st) establishment of the topic and guiding question; 2nd) establishment of inclusion and exclusion criteria; 3rd) identification of studies; 4th) categorization of studies; 5th) analysis and interpretation of results; 6th) synthesis of knowledge and presentation of results.

PICO, which stands for Patient, Intervention, Comparison, and Outcomes, was the strategy used to establish the guiding question. These four components are essential to establish the guiding question and, consequently, seek scientific evidence^16^.

Therefore, the topic chosen was the acquisition of microbiota among newborns according to the route of birth delivery. The guiding question was: What is the production of knowledge concerning the association between the acquisition of microbiota among newborns and the route of birth delivery? The first element of the strategy (P) refers to newborns; the second (I) to C-section; the third refers to vaginal delivery (C); and the fourth element (O) refers to newborns’ acquired microbiota.

Inclusion criteria were: papers published in Portuguese, English, or Spanish; answering the guiding question; with up to ten years since publication. Exclusion criteria were letters, editorials, experts’ opinions, reviews, and studies addressing premature infants, or not comparing the route of birth nor addressing newborns.

An advanced search was performed in the following electronic databases to select the studies for this review: Medical Literature Analysis and Retrieval System Online (MEDLINE) (via PubMed), Latin American and Caribbean Health Sciences Literature (LILACS), and *Índice Bibliográfico Español en Ciencias de la Salud* (IBECS) (via Virtual Health Library-VHL), on August 10th, 2020. The following descriptors were used in the search strategy: ((“MICROBIOTA”) AND ((“DELIVERY, OBSTETRIC”) OR (“CESAREAN SECTION”) OR (“PARTURITION”)) NOT (“MILK, HUMAN”)) in the PubMed and (tw:(MICROBIOTA)) AND (tw:(HUMANS)) AND (tw:(PARTURITION OR CESAREAN SECTION OR DELIVERY, OBSTETRIC)) AND NOT (tw:(HUMAN, MILK)) in the VHL.

The equivalent descriptors in Portuguese and Spanish were used in the VHL to identify Latin American studies, namely: (tw:(MICROBIOTA)) AND (tw:(HUMANOS)) AND (tw:(PARTO OR CESÁREA OR PARTO OBSTÉTRICO)) AND NOT (tw:(LEITE HUMANO OR LECHE HUMANA)).

The descriptors used in the PubMed were selected from the Medical Subject Headings (MeSH terms), used for the efficient indexing of publication in the topic^17^. The descriptors from the Health Science Descriptors (DeCS) were used in the search in the VHL.

The Preferred Reporting Items for Systematic Reviews and Meta-Analyses - PRISMA was adapted to guide this review’s report^18^. After gathering the studies identified in the two electronic databases, those that appeared more than once were excluded after comparing titles, authors, year of publication, and country of origin. Duplicated studies were kept in the database with the largest number of references (PubMed). Two of the authors independently screened the titles and abstracts according to the eligibility criteria.

Afterward, the full texts of the studies selected were considered in a new screening process. The researchers independently examined all the publications to choose the studies that met the inclusion criteria previously mentioned. Conflicts were resolved in a consensual meeting.

The studies were then analyzed using an instrument to collect data^19^ addressing the study’s title, journal, authors, country of origin, language, year of publication, hosting institution, type of scientific journal, methodological characteristics, and an assessment of the studies’ methodological rigor. Data were entered in the Excel program.

The studies were also analyzed according to the level of evidence. In this study, the level of evidence was organized into seven levels: Level I - systematic review or meta-analysis of randomized controlled clinical trials; Level II - evidence obtained from at least one well-designed randomized controlled clinical trial; Level III - well-designed controlled trial without randomization; Level IV - evidence from well-designed case-control or cohort studies; Level V - systematic reviews of descriptive and qualitative studies; Level VI - evidence form a single descriptive or qualitative study; Level VII - evidence from the opinion of authorities and/or reports of expert committees^20^.

The studies were tabulated and rigorously analyzed, interpreted, and synthesized. Two thematic categories were chosen according to the topics addressed in the studies to interpret the results.

## Results

**Figure 1:**
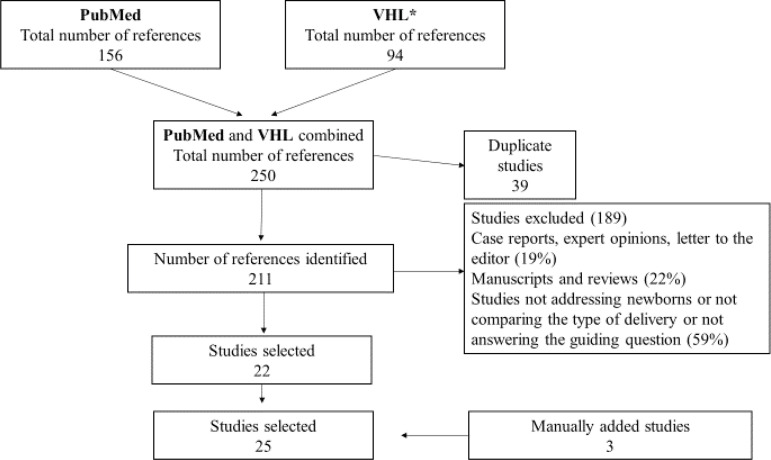
Studies selection process: adapted from the PRISMA flowchart^(^ 18 ^)^

This study’s sample was composed of 25 studies. The entire process of searching, excluding, and selecting studies is described in detail in a flowchart (Figure 1).

The year of publication among the studies selected ranged from 2010 and 2019: 32% of the studies were published in 2016, followed by 12% of the publications published in 2018, 2017, and 2015. Regarding the country of origin, the United States presented the highest number of studies (20%), followed by Sweden and China, with 16% each. All the papers were written in English, and the periodical with the highest number of publications was PloS One with three papers. Nature Medicine, Nat Commun, EBioMedicine, Journal of Allergy and Clinical Immunology, Journal of Pediatric Gastroenterology and Nutrition, and Scientific Reports published two papers each.

Analysis of the studies revealed similar results and discussions. Hence, two analytical categories emerged from the studies’ analysis: microbiota and immune system and route of birth delivery and neonatal microbiota. Both are presented in the discussion section.

Figure 2 presents a synthesis of the knowledge presented by the studies with information concerning the authors, study designs, level of evidence, and main results.

**Figure 2:** Synthesis of the knowledge presented by the studies selected

Authors	Year	Study design/Level of evidence	Main Results
Liu, et al.^(^ 21 ^)^	2019	Well-designed controlled trial without randomization/III	Only samples of the amniotic fluid showed significant differences (p<0.001) according to the type of birth. While meconium, placenta, and fetal membrane samples showed no significant differences between newborns born vaginally or via C-section. Vaginal delivery: *Lactobacillus* and *Gardnerella*; C-section: *Thermus* and *Tepidiphilus*.
Reyman, et al.^(^ 22 ^)^	2019	Cohort/IV	Up to two months of life, the microbial community of children born vaginally was more stable than that of children born via C-section. Abundant *Bifidobacteria* is associated with the type of delivery, age, and whether the child is breastfed. Breastfeeding, however, does not make up for the lack of *Bifidobacteria* among children born via C-section. Vaginal delivery: *Bifidobacteria* and *Escherichia*; C-section: *Klebsiella* and *Enterococcus*.
Li, et al.^(^ 23 ^)^	2018	Well-designed controlled trial without randomization/III	The vaginal delivery group has significantly more *Bifidobacteria* and *Akkermansiaceae*, showing the beneficial aspect of natural birth. Two common pathogenic bacteria, Providencia and Gardnerella, were also found in some of the infants in this group, which may be explained by the presence of mothers with vaginal infections that had not been manifested.
Shi, et al.^(^ 24 ^)^	2018	Cohort/IV	The microbiome of children born vaginally is a little more diverse than that of children born via C-section. Vaginal delivery: *Actinobacteria, Gammaproteobacteria*, and *Betaproteobacteria*; C-section: *Deinococcus, Alphaproteobacteria*, and *Bacilli*.
Wampach, et al.^(^ 25 ^)^	2018	Cohort/IV	Type of delivery was considered the dominant driver of the colonization of the neonatal gut microbiome. Vaginal delivery influences the transference of functional characteristics involved in microbial pathways, such as the biosynthesis of lipopolysaccharides that are important in developing a newborn's immune system.
Brazier, et al.^(^ 26 ^)^	2017	Well-designed controlled trial without randomization/III	The type of delivery influenced the fecal microbial composition. Vaginal delivery: *Bacteroides* and *Collinsella*; C-section: *Klebsiella* and *Sarcina*.
Chu, et al.^(^ 27 ^)^	2017	Cohort/IV	The neonatal microbial community structure at the time of delivery did not show significant differences according to the body's site. Vaginal delivery: *Lactobacillus*; C-section: *Propionibacterium* and *Streptococcus*. The microbiota of newborns born vaginally tended to be similar to their mothers' vagina, while infants born via elective C-sections were mainly populated by the microbiota found on their mothers' skin.
Hill, et al.^(^ 28 ^)^	2017	Cohort/IV	The structure of an infant's gut microbiota is affected by the type of delivery. Vaginal birth: *Bifidobacteria, Bacteroides*; C-section: *Clostridium*. A large diversity of individual population structures was found within each group, showing the heterogeneous gut microbiota composition of developing infants.
Bokulich, et al.^(^ 29 ^)^	2016	Well-designed controlled trial without randomization/III	The children born via C-section showed a significantly greater phylogenetic diversity (p<0.05). However, these significantly decreased during the first month after birth while these children subsequently presented a lower diversity and less richness up to 2 years of age.
Bosch, et al.^(^ 30 ^)^	2016	Cohort/IV	Children born vaginally tend to change profiles dominated by *Moraxella* and *Corynebacterium/Dolosigranulum* in an earlier stage than children born via C-section, who remain longer in a profile dominated by S. aureus.
Brumbaugh, et al.^(^ 31 ^)^	2016	Well-designed controlled trial without randomization/III	Significant differences were found at the phylum level and bacterial abundance at a gender level, according to the type of delivery for all the infants' samples. Vaginal delivery: Oropharynx: Firmicutes (*Lactobacillus*). Fecal samples: Bacteroidetes; C-section: Oropharynx: Actinobacteria (*Propionibacterium*) and *Proteobacteria*. Fecal samples: *Proteobacteria*. Disturbances in colonization and succession within the human gut in early life may influence the risk of long-term illnesses.
Dominguez-Bello, et al.^(^ 12 ^)^	2016	Well-designed controlled trial without randomization/III	Regardless of the body site, the microbiome of infants born vaginally or via C-section were more similar to their mothers' vaginal microbiome, when exposed to vaginal fluid, than that of infants born via C-section but not exposed.
Kristensen; Herinksen^(^ 32 ^)^	2016	Cohort/IV	C-section was associated with infection and inflammation of the mucosa. The effect of elective C-sections was more significant for asthma than emergency C-sections. Estimates of respiratory tract disease did not change after adjusting for neonatal respiratory morbidity.
Martin, et al.^(^ 33 ^)^	2016	Cross-sectional/VI	The type of delivery was one of the factors that strongly impacted the initial composition of newborns' microbiota. The infants born vaginally had significantly higher bacterial counts in the meconium than those born by C-section. Infants born via C-section had late colonization of various groups of bacterial species. Vaginal birth: *Bifidobacterium*, *Bacteroides fragilis, B. ovatus, B. vulgatus, B. uniformis, B. caccae, and B. longum subsp. Longum; C-section: Enterococcus and C. perfringens*.
Shilts, et al.^(^ 34 ^)^	2016	Cohort/IV	The taxonomic profile of the nasal microbiome of infants born vaginally was closer to the nasal microbiome previously found in adults, compared to infants born via C-section. This finding supports the hypothesis that the nasal microbiome of infants born vaginally may represent an environment successfully colonized by stable commensal microbiota. Vaginal delivery: Actinobacteria (*Corynebacterium*); C-section: Firmicutes (*Staphylococcus*).
Stokholm, et al.^(^ 35 ^)^	2016	Cohort/IV	Type of birth was associated with different gut bacterial colonization patterns in early childhood, which normalizes during the first year of life. Elective and emergency C-sections were associated with distinctly different colonization patterns. Vaginal birth: E. coli; C-section: *Citrobacter freundii, Enterobacter cloacae, Enterococcus faecalis, Klebsiella oxytoca, Klebsiella pneumoniae,* and *Staphylococcus aureus*.\
Bäckhed, et al.^(^ 36 ^)^	2015	Cohort/IV	The route of birth strongly affected the microbiome species in neonates. Vaginal birth: Bacteroides, Bifidobacterium, Parabacteroides, *Escherichia/Shigella*; C-section: *Enterobacter sp., Haemophilus sp., Staphylococcus sp., Streptococcus australis,* and *Veillonella sp*.
Dogra, et al.^(^ 37 ^)^	2015	Cohort/IV	The bacteria colonizing newborns' bodies promote a long-lasting effect in the immune system or on the gut barrier function, driven by the type of delivery. Vaginal birth: *Bifidobacterium* and *Collinsella*. C-section: *Klebsiella, Enterobacteriaceae,* and *Streptococcus*. A lower or later colonization by *Bifidobacteria* was found among infants born via C-section; *Bifidobacteria* are considered to be ideal in a newborn's organism.
Dong, et al.^(^ 38 ^)^	2015	Cross-sectional/VI	The route of the birth delivery had a more significant impact on the gut microbiota structure than on its diversity during the first 4 days of life. Vaginal birth: DAY 2: *E coli* and *Bacteroides sp*. DAY 4: *Bifidobacterium sp* and *Bacteroides sp*; C-section: DAY 2: *Staphylococcus sp, Clostridium sp*, and *Enterobacter sp*. DAY 4: *Clostridium sp* and *Streptococcus sp*.
Hesla, et al.^(^ 39 ^)^	2014	Cohort/IV	Vaginal birth: *Bacteroides*. C-section: *Enterobacteriaceae, Clostridium, Haemophilus, Veillonella*.
Jakobsson, et al.^(^ 40 ^)^	2014	Well-designed controlled trial without randomization/III	A lower microbial diversity was found among infants born via C-section and lower circulating levels of the Th1-type chemokines, CXCL10 and CXCL11.
Makino, et al.^(^ 41 ^)^	2013	Well-designed controlled trial without randomization/III	The number of *Bifidobacteria* was significantly lower among infants born through C-section than among infants born vaginally up to 7 days of age. *Bifidobacteria* gut colonization is considered to begin more rapidly among infants born vaginally than among infants born through C-sections.
Pandey, et al.^(^ 42 ^)^	2012	Well-designed controlled trial without randomization/III	The initial colonization and acquisition of gut microbiota may strongly influence the status of cellular and humoral elements of the immune system of the gut mucosa during a newborn's life. Vaginal delivery: *Staphylococcus haemolyticus; Acinetobacter sp., Bifidobacterium sp. C-section: Roseomonas pecuniae, Paracoccus sp., Enterococcus sp., Streptococcus vestibularis, Chryseomicrobium imtechense, and Staphylococcus sp., Clostridium difficile, Citrobacter sp.* and *Escherichia coli*.
Biasucci, et al.^(^ 43 ^)^	2010	Well-designed controlled trial without randomization/III	The study reports *Bifidobacterium's* presence in 13 of the 23 (56.5%) samples from infants born vaginally, but not in the samples obtained from infants born via C-section.
Dominguez-Bello, et al.^(^ 44 ^)^	2010	Randomized Clinical Trial/I	The newborns harbored bacterial communities that were essentially undifferentiated on the skin, nasal, nasopharynx, and gut habitats, regardless of the type of birth delivery. These results show that human microbiota is homogeneously distributed throughout the body in the early stage of community development.

## Discussion

A rise in the C-section rates, diet changes, the indiscriminate use of antibiotics, and antimicrobial agents have altered the natural microbial composition of the human body. These may disturb the balance of commensal organisms and alter their metabolic network, favoring the growth of potentially pathogenic constituents, negatively reflecting on the population’s health^23^^,^ 45 ^-^ 46.

Therefore, it is essential to understand the importance of the microbiota for the immune system and its relationship with the route of birth delivery and a newborn’s microbiota composition.

Studies show that microbiota is a crucial and active inducer of regulatory responses of the immune system^11^^,^ 47. It is capable of inducing Regulatory T cells (TReg) and macrophages to act against pathogenic antigens^48^. Dysbiosis may prolong immunological immaturity and, consequently, increase the risk of autoimmune disorders^11^^,^ 34 ^,^ 40 ^,^ 49 ^-^ 54.

The neonatal period is considered a critical window in the development of the immunological system. Appropriate microbial stimulation is essential for the appropriate maturation of TReg cells response^55^. Short-chain fatty acids, a by-product of bacterial fermentation, are known for modulating T cells’ regulatory homeostasis (TReg)^56^. These are responsible for immunomodulatory or immunosuppressive responses; that is, they play a role in the body’s immune tolerance, controlling inflammatory responses^57^. Dysregulation of these cells is directly linked to the development of allergic problems and autoimmune diseases^42^^,^ 48.

Additionally, the human organism’s microbiota limits the colonization of pathogens based on a competition for metabolites, a process known as “colonization resistance”^57^^-^ 58. The colonizing microbiota establishes a homeostatic relationship with the host, in which the colonization microorganisms benefit from the nutrient-rich environment, ensuring their survival and providing their hosts with a greater ability to absorb nutrients from food, establishing networks and biofilms capable of protecting the organism from pathogenic antigens, among other benefits^45^^,^ 48.

*Bifidobacterium* and *Lactobacillus* are considered the ideal bacteria for the human body because they are commensal bacteria^22^^,^ 44 ^,^ 52 ^,^ 59. The early presence of *Bifidobacterium* between one week and three months of age was associated with a lower risk of developing eczema^59^^-^ 60. Additionally, a decreased number of *Bifidobacterium* and *Lactobacillus* seem to be related to the emergence of allergies^42^^,^ 52 ^,^ 59. Some studies do not report significant differences between the microbiota of asthmatic and healthy individuals; however, low levels of *Bifidobacterium* are found in the long run among individuals with asthma, compared to recently diagnosed individuals^61^.

Another component found in the microbiota of newborns is *Bacteroides*. Some of them help regulate gut immunity, and a deficiency of these bacteria was identified among infants born via C-section. This situation causes the rupture of tolerogenic response, contributing to inflammation and obesity^37^^,^ 42. Additionally, these bacteria are involved in the digestion of human milk, and scarcity of these in the neonatal gut may cause digestive problems^26^^,^ 28.

These claims are explained based on the Old Friends theory. Commensal microorganisms train the immunological system to develop tolerance mechanisms as a strategy for their own survival. Hence, the immune system recognizes and eliminates harmful bacteria but does not react against useful species or the organism itself^60^^,^ 62. Old Friends bacteria can activate the production of anti-inflammatory cytokines, differentiating themselves from pathogens that promote pro-inflammatory cytokines^52^^,^ 62.

Regarding the route of birth delivery and neonatal microbiota, the first influences the acquisition and colonization of bacteria in newborns’ bodies^22^^-^ 44 ^,^ 52 ^,^ 63, especially during early childhood, usually stabilizing in the first year of life^35^. An infant’s immune system is exposed for the first time to microorganisms during vaginal delivery or C-section^12^.

Many of an infant’s body sites come into contact with the environment’s microbiota, and colonization occurs. The studies analyzed the neonatal microbial colonization in various body sites, and the gut was the site most frequently studied using fecal samples^12^^,^ 21 ^-^ 22 ^,^ 24 ^-^ 26 ^,^ 28 ^,^ 31 ^,^ 33 ^,^ 36 ^-^ 37 ^,^ 40 ^-^ 41 ^,^ 43 ^-^ 44. Others studies analyzed the skin^12^^,^ 27 ^,^ 44, and oral and nasal mucosa^12^^,^ 21 ^,^ 27 ^,^ 30 ^-^ 32 ^,^ 34 ^-^ 35 ^,^ 44. However, it is argued that bacterial communities show a homogeneous distribution pattern in the most diverse body habitats; i.e., regardless of the route of birth, microbial colonization shows little or no differences between body sites^27^^,^ 42.

Infants born vaginally have a higher concentration of *Bacteroides, Bifidobacterium,* and *Lactobacillus* in the first days of life, and a greater microbial variability occurs in the following weeks^22^^,^ 33 ^,^ 43 ^,^ 47 ^,^ 62. These infants’ microbiota is more similar to their mothers’ vaginal microbiome^21^^-^ 22 ^,^ 27 ^,^ 44 ^,^ 64. Note that the main bacterial communities found in mothers’ vaginas are *Lactobacillus*, *Bifidobacterium*, and *Streptococcus* 65 ^-^ 66, though there are controversies in the literature. The vaginal flora composition during pregnancy is relatively less diverse compared to that of non-pregnant women. This is due to a decreased number of some members of the vaginal community and enrichment of *Lactobacillus* target species. Hence, the composition of a pregnant woman’s vaginal microbiota is more stable, conferring a protective role against ascending infections^65^.

Infants born via C-section present a microbiome similar to the maternal skin and the hospital environment^47^^,^ 56 ^,^ 62 ^-^ 63, which is also less diverse, mainly composed of *Staphylococcus, Streptococcus,* and *Clostridium*. Although, a longitudinal study^29^ addressing 43 infants reports a significantly greater bacterial diversity among those born via C-section than those born vaginally. This variety, however, decreased from the first month of life up to two years of age while these children presented less phylogenetic richness compared to children born vaginally^29^.

The *Staphylococcus, Clostridium, Klebsiella, Enterobacter,* and *Enterococcus* species were more frequently found among infants born via C-section. These bacteria are resistant to various antibiotics and are endemic in the hospital setting^22^^-^ 23 ^,^ 25 ^-^ 26 ^,^ 35 ^,^ 44. Studies suggest that C-sections cause effects similar to the use of antibiotics, changing the maturation patterns of microbiota in neonates^29^.

On the other hand, the effects of elective and emergency C-sections differ^35^. Elective C-sections imposed a greater risk of asthma than emergency C-sections^32^. Infants born via emergency C-section undergo the effects of labor and, for this reason, have a bacterial composition similar to that of infants born vaginally^12^. Consequently, these children are less likely to develop an immune system problem compared to infants born via elective C-sections.

The microbiota of healthy adults is stable and diversified^67^. Aberrant and scarce microbiota are related to various health disorders^40^^,^ 42 ^,^ 52 ^,^ 54. Additionally, the low diversity of colonizing bacteria is related to a larger number of antibody-secreting cells, causing exaggerated immune responses^67^. In this sense, the immune system is believed to have a more beneficial response to the health-disease continuum in the presence of a diverse and stable microbiota.

Despite the systematized search and selection of studies, it is worth mentioning that it is impossible to exhaust the entire literature addressing this topic. Therefore, this study’s limitations include the search method, which was restricted to only two databases, the 10-year timeframe, and the fact that only studies written in Portuguese, English, or Spanish (the languages the authors are proficient) were selected.

Notwithstanding its limitations, this study presents evidence of 25 studies, most of which were clinical trials and cohort studies. It clearly shows that the route of birth influences an infant’s microbiota, and it is assumed that it may compromise infants’ health, influencing the development of allergies, immunological/metabolic diseases, and obesity. Thus, this study contributes to the advancement of scientific knowledge and reinforces the importance of policies and practices intended to discourage C-sections worldwide, but especially in Brazil.

## Conclusion

Microbial colonization during the fetal period, intrapartum, and after birth is crucial in the host-microbial mutualism; the primary function is the maturation and development of newborns’ immune systems. The maternal vaginal microbiota provides newborns with a greater variety of colonizing microorganisms, responsible for training and adapting the infants’ immune system. Therefore, it is clear that vaginal delivery is ideal, and only in the presence of medical indications should a C-section be endeavored.

Currently, the Brazilian obstetrical service presents high rates of C-sections. The implications of using this route of birth delivery involve increased fetal, neonatal, and maternal morbidity and mortality. In this sense, this study supports the choice of the route of birth delivery and, consequently, favors a decrease in unnecessary C-sections. It is crucial to provide information to pregnant women and/or couples and health workers to make informed and educated decisions.

Research addressing the role of microbiota in human health and its association with the route of birth tends to increase, and further studies are crucial to understanding the complex interaction between one’s microbiota and the immune system while the route of birth delivery influences both. Thus, there are gaps concerning the mechanisms involved in the training and maturation of newborns’ immune systems and its implications in adulthood.
